# Establishment of C_20_Mab-11, a novel anti-CD20 monoclonal antibody, for the detection of B cells

**DOI:** 10.3892/ol.2020.11753

**Published:** 2020-06-17

**Authors:** Yoshikazu Furusawa, Mika Kato Kaneko, Yukinari Kato

**Affiliations:** 1 Department of Antibody Drug Development, Tohoku University Graduate School of Medicine, Sendai, Miyagi 980-8575, Japan; 2 New Industry Creation Hatchery Center, Tohoku University, Sendai, Miyagi 980-8575, Japan

**Keywords:** CD20, monoclonal antibody, western blotting, flow cytometry, immunohistochemistry

## Abstract

CD20 is one of several B-lymphocyte antigens that has been shown to be an effective target for the detection and treatment of B-cell lymphomas. Sensitive and specific monoclonal antibodies (mAbs) are required for every application used for the diagnosis of B-cell lymphoma. Although many anti-CD20 mAbs have been established, the types of applications, those anti-CD20 can be used in, are limited. In this study, we aimed to establish novel anti-CD20 mAbs to be used for broad applications, such as flow cytometry, western blot, and immunohistochemical analyses, using the Cell-Based Immunization and Screening (CBIS) method. One of the established mAbs, C_20_Mab-11 (IgM, kappa), detected overexpression of CD20 in CHO-K1 or LN229 cell lines, indicating that C_20_Mab-11 is specific for CD20. In western blot analyses, C_20_Mab-11 detected not only overexpression of CD20 in CHO-K1 or LN229 cell lines, but also CD20 of BALL-1 and Raji cells with both sensitivity and specificity. Furthermore, C_20_Mab-11 strongly stained B cells of the lymph follicle and B cell lymphomas in immunohistochemical analyses. These results indicate that C_20_Mab-11 develped by CBIS method, is useful for the detection of CD20 in lymphoma tissues by flow cytometry, western blot, and immunohistochemical analyses and potentially could be beneficial for the treatment of B cell lymphomas.

## Introduction

CD20 is an integral membrane protein with a molecular weight of 33–37 kDa, which is expressed in high densities only on B lymphocytes (1,2). CD20 has four membrane-spanning domains and consists of 297 amino acids (aa). The two extracellular domains are located at 72–80 and 142–182 aa. Homo-oligomerization of CD20 into tetramers functions as a calcium channel. CD20 is found expressed on B cells from pre-B to mature B cell development and it is also detected on many kinds of non-Hodgkin lymphomas (NHL) (3). CD20 is detected on 50% of B-lymphoblastic leukemia/lymphoma (B-ALL/LBL) originating from pre-B cells, but it is not detected in terminally differentiated plasma cell malignancies (4,5).

The development of sensitive and specific monoclonal antibodies (mAbs) are critical for diagnosis and chemotherapeutic treatment for many types of cancer (6). However, the production of sensitive and specific mAbs is known to be a very difficult procedure because it requires detecting the extracellular loop of multi-pass transmembrane proteins (7). As mentioned above, CD20 possesses two extracellular transmembrane loops that are very small in size. Therefore, compared to the production of mAbs against single-pass transmembrane proteins, such as CD44 (8) or PD-L1 (9), it is much more difficult to develop sensitive and specific anti-CD20 mAbs for use in multiple applications.

In our previous studies, we developed the Cell-Based Immunization and Screening (CBIS) method, in which cell lines are exclusively used for both immunization and screening. CBIS has previously been used for developing several mAbs against various proteins, including the five transmembrane protein CD133 (7). Using the CBIS method, we have successfully produced sensitive and specific mAbs useful for not only flow cytometry, but also western blot and immunohistochemical analyses (7–17).

We have two objectives for developing anti-CD20 mAbs in this study. First, we employed CBIS to establish novel anti-CD20 mAbs with a focus on improving this method for developing advantageous mAbs against multiple-pass transmembrane proteins. Second, we aimed to develop multi-use anti-CD20 mAbs that can be used for flow cytometric, western blot, and immunohistochemical analyses.

## Materials and methods

### 

#### Cell lines

P3X63Ag8U.1 (P3U1), Chinese hamster ovary (CHO)-K1, Lec1, Lec2, Lec8, and LN229 cells were obtained from the American Type Culture Collection. Raji and BALL-1 were obtained from the Cell Resource Center for Biomedical Research (Institute of Development, Aging and Cancer, Tohoku University, Miyagi, Japan). DNA encoding the CD20 gene (IRAL012D02) was provided by the RIKEN BRC through the National BioResource Project of MEXT, Japan. The open reading frame of CD20 plus an N-terminal PA tag was subcloned into a pCAG-Neo or pCAG-Ble vector (FUJIFILM Wako Pure Chemical Corporation). CHO/CD20 and CHO/mock were produced by transfecting pCAG-Neo/CD20 and pCAG-Neo into CHO-K1 cells, respectively, using a Gene Pulser Xcell electroporation system (Bio-Rad Laboratories, Inc.). LN229/CD20 and LN229/mock were produced by transfecting pCAG-Ble/CD20 and pCAG-Ble into LN229 cells, respectively, using a Neon transfection system (Thermo Fisher Scientific, Inc.). Lec1/CD20 (*N*-glycan-deficient), Lec2/CD20 (sialic acid-deficient), and Lec8/CD20 (galactose-deficient) were produced by transfecting pCAG-Ble/CD20 into Lec1, Lec2, and Lec8 cells, respectively, using a Neon transfection system. Lec1/mock, Lec2/mock, and Lec8/mock were produced by transfecting pCAG-Ble into Lec1, Lec2, and Lec8 cells, respectively, using a Neon transfection system. The cell line BALL-1/CD20-KO (BINDS-24) was generated by transfecting CRISPR/Cas9 plasmids for CD20 (Thermo Fisher Scientific, Inc.) using a Neon transfection system. Stable transfectants were established using SH800 (Sony Corp.).

P3U1, CHO-K1, CHO/CD20, CHO/mock, Lec1, Lec1/CD20, Lec1/mock, Lec2, Lec2/CD20, Lec2/mock, Lec8, Lec8/CD20, Lec8/mock, Raji, BALL-1, and BINDS-24 were cultured in Roswell Park Memorial Institute (RPMI) 1640 medium (Nacalai Tesque, Inc.). LN229, LN229/CD20, and LN229/mock were cultured using Dulbecco's modified Eagle's medium (DMEM; Nacalai Tesque, Inc.). The media were supplemented with 10% heat-inactivated fetal bovine serum (Thermo Fisher Scientific Inc.), 100 units/ml of penicillin, 100 µg/ml of streptomycin, and 25 µg/ml of amphotericin B (Nacalai Tesque, Inc.). L-proline (0.04 mg/ml; MP Biomedicals, LLC) was added to Lec1, Lec2, and Lec8. The cells were grown in an incubator at 37°C with humidity and 5% CO_2_ and 95% air atmosphere.

#### Reverse transcription-PCR (RT-PCR)

Total RNAs were prepared from cell lines using an RNeasy mini prep kit (Qiagen Inc.). The initial cDNA strand was synthesized using SuperScript IV Reverse Transcriptase (Thermo Fisher Scientific, Inc.) by priming nine random oligomers and an oligo(dT) primer according to the manufacturer's instructions. We performed 35 cycles of PCR for amplification using HotStarTaq DNA Polymerase (Qiagen Inc.) with 0.2 mM of primer sets: CD20 sense (5′-ATGACAACACCCAGAAATTC-3′), CD20 antisense (5′-TTAAGGAGAGCTGTCATTTTC-3′), GAPDH sense (5′-CAATGACCCCTTCATTGACC-3′), and GAPDH antisense (5′-GTCTTCTGGGTGGCAGTGAT-3′).

#### Animals

All animal experiments were performed in accordance with relevant guidelines and regulations to minimize animal suffering and distress in the laboratory. Animal experiments described in the hybridoma production were approved by the Animal Care and Use Committee of Tohoku University (Permit no: 2016MdA-153). Mice were monitored for health every day. The duration of the experiment was four weeks. A body weight loss exceeding 25% of total body weight were defined as a humane endpoint. Mice were euthanized by cervical dislocation, and the death was verified by respiratory arrest and cardiac arrest.

#### Hybridoma production

Two female BALB/c mice (6 weeks old) were purchased from CLEA Japan. Animals were housed under specific pathogen-free conditions. Animal experiments described in the hybridoma production were approved by the Animal Care and Use Committee of Tohoku University (Permit no: 2016MdA-153). Briefly, LN229/CD20 cells (1×10^8^ cells) were immunized into two BALB/c mice using intraperitoneal injection together with Imject Alum (Thermo Fisher Scientific, Inc.). After three additional immunizations, a booster injection was administered two days before harvesting spleen cells. These spleen cells were fused with P3U1 cells using polyethylene glycol 1500 (Roche Diagnostics), and then hybridomas were grown in an RPMI medium supplemented with sodium hypoxanthine, aminopterin, and thymidine (Thermo Fisher Scientific, Inc.). The culture supernatants were used for hybridoma screening by flow cytometry.

#### Flow cytometry

Cells were harvested by brief exposure to 0.25% trypsin with 1 mM ethylenediaminetetraacetic acid (Nacalai Tesque, Inc.). After washing with phosphate buffered saline (PBS) containing 0.1% bovine serum albumin (BSA), the cells were treated with 10 µg/ml of C_20_Mab-11 for 30 min at 4°C, followed by treatment with Alexa Fluor 488-conjugated anti-mouse IgG (1:2,000; Cell Signaling Technology, Inc.). Fluorescence data were collected using Spectral Cell Analyzer SA3800 (Sony Corp.).

#### Western blot analysis

Cell lysates were prepared by 1% Triton X-100 and cell debris was removed by centrifugation. Cell lysates were boiled in sodium dodecyl sulfate sample buffer with a reducing reagent (Nacalai Tesque, Inc.). These proteins (10 µg) were electrophoresed on 5–20% polyacrylamide gels (FUJIFILM Wako Pure Chemical Corporation) and transferred onto polyvinylidene difluoride membranes (Merck KGaA). After blocking with 4% skim milk (Nacalai Tesque, Inc.), membranes were incubated with 10 µg/ml of C_20_Mab-11, 1 µg/ml of NZ-1 (anti-PA tag) or 1 µg/ml of anti-β-actin for control (clone AC-15; Sigma-Aldrich Corp.), followed by incubation with secondary antibody peroxidase-conjugated anti-mouse immunoglobulin (1:1,000; Agilent Technologies Inc., Santa Clara, CA, USA) or anti-rat IgG (1:10,000; Sigma-Aldrich Corp.), respectively. Finally, proteins were detected with ImmunoStar LD (FUJIFILM Wako Pure Chemical Corporation) using the Sayaca-Imager (DRC Co. Ltd.).

#### Immunohistochemical analyses

One formalin-fixed paraffin-embedded (FFPE) tissue sample from an oropharyngeal squamous cell carcinoma patient who underwent surgery at Sendai Medical Center was used for this study (13). Written informed consent was obtained from the patient for sample procurement and subsequent data analyses. Tissue microarray (CC00-10-001) including lymphomas, normal lymph node, and normal thyroid was purchased from Cybrdi, Inc.

The 4-µm thick paraffin-embedded tissue sections were directly autoclaved in EnVision FLEX Target Retrieval Solution High pH (Agilent Technologies, Inc.) for 20 min. After blocking with the SuperBlock T20 (PBS) Blocking Buffer (Thermo Fisher Scientific, Inc.), tissue sections were incubated with C_20_Mab-11 (5 µg/ml) for 1 h at room temperature and treated with the Envision+ Kit for mouse (Agilent Technologies, Inc.) for 30 min. Color was developed using 3,3′-diaminobenzidine tetrahydrochloride (Agilent Technologies, Inc.) for 2 min, and counterstaining was performed using hematoxylin (FUJIFILM Wako Pure Chemical Corporation).

#### Statistical analysis

Statistical analysis was conducted using t-test with GraphPad Prism 6 (GraphPad Software, Inc.). P<0.05 was considered to indicate a statistically significant difference. All data are expressed as the mean ± SEM.

## Results

### 

#### Establishment of anti-CD20 monoclonal antibodies

**Figure 1.**
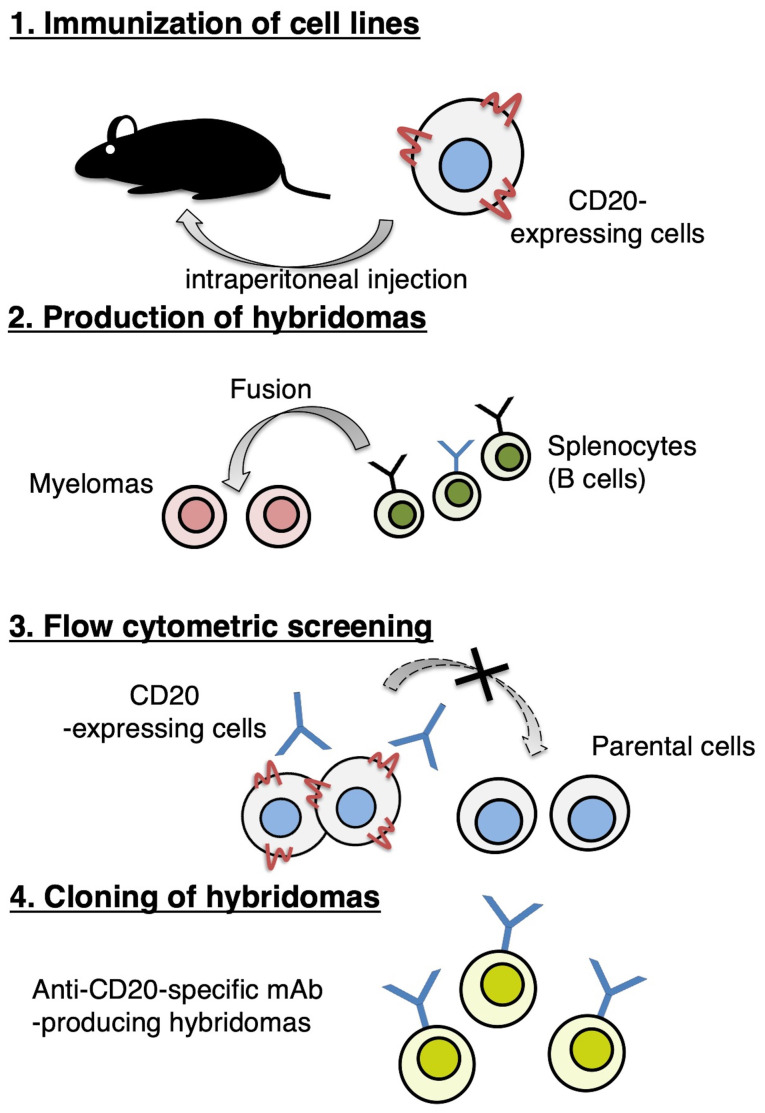
Production of anti-CD20 monoclonal antibodies. Procedure of Cell-Based Immunization and Screening method. LN229/CD20 cells were immunized into BALB/c mice using intraperitoneal injection. Screening was performed using flow cytometry.

In this study, we employed the CBIS method (Fig. 1). First, we immunized two mice with LN229/CD20 cells and their spleen cells were harvested and grown as hybridomas in cell culture. Next, supernatants from cultured hybridomas positive for CHO/CD20 and negative for CHO-K1, were selected by flow cytometry. We detected strong signals from CHO/CD20 and weak or no signals from CHO-K1 in 14 of the 960 wells (1.5%). Further screening using western blotting and immunohistochemistry techniques led to the establishment of C_20_Mab-11 (IgM, kappa).

**Figure 2.**
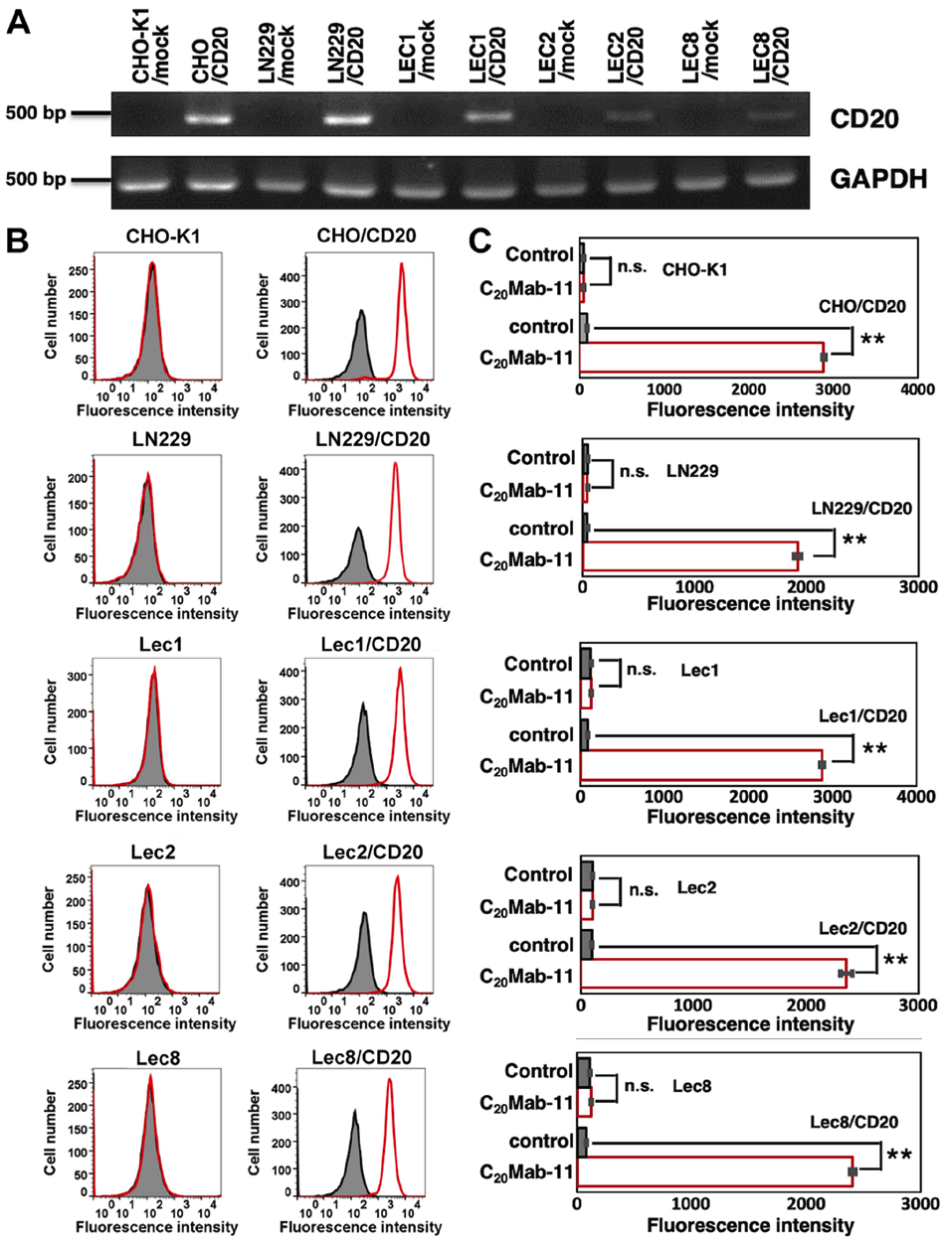
Detection of CD20 by C_20_Mab-11 using flow cytometry. (A) Reverse transcription-PCR for CD20. Overexpression of CD20 was confirmed in CHO/CD20, LN229/CD20, Lec1/CD20, and Lec2/CD20, and Lec8/CD20 cell lines. GAPDH was used as an internal control. (B) Detection of CD20 by C_20_Mab-11. CHO-K1, CHO/CD20, LN229, LN229/CD20, Lec1 (*N*-glycan-deficient), Lec1/CD20, Lec2 (sialic acid-deficient), Lec2/CD20, and Lec8 (galactose-deficient), and Lec8/CD20 cells were treated with C_20_Mab-11 (red line) at a concentration of 10 µg/ml or 0.1% bovine serum albumin in PBS (gray) for 30 min, followed by incubation with secondary antibodies. (C) Fluorescence intensity was quantified. **P<0.01. n.s., not significant. All data are presented as the mean ± SEM.

CD20 expression was investigated by RT-PCR. CD20 was not detected in CHO-K1/mock, LN229/mock, Lec1/mock, Lec2/mock, and Lec8/mock (Fig. 2A). Then, overexpression of CD20 was confirmed in CHO/CD20, LN229/CD20, Lec1/CD20, and Lec2/CD20, Lec8/CD20. We used these stable transfectants of CD20 in flow cytometry and western blot analyses to characterize C_20_Mab-11.

#### Flow cytometric analyses

Using flow cytometry analysis, we found that C_20_Mab-11 reacted with CHO/CD20 cells, but not with CHO-K1 cells (Fig. 2B). Similarly, C_20_Mab-11 reacted with LN229/CD20 cells, but not with LN229 cells. Additionally, we performed flow cytometry using CD20-stable transfectants of glycan-deficient CHO cell lines (Lec1, Lec2, and Lec8), although it was previously reported that CD20 is not glycosylated (18). C_20_Mab-11 reacted with Lec1/CD20 (*N*-glycan-deficient), Lec2/CD20 (sialic acid-deficient), and Lec8/CD20 (galactose-deficient) cells, but not with Lec1, Lec2, and Lec8 cells, indicating that the binding epitope of C_20_Mab-11 is independent of glycans. These results indicate that C_20_Mab-11 is specific for CD20. Fluorescence intensity was quantitatively analyzed (Fig. 2C).

#### Western blot analysis

**Figure 3.**
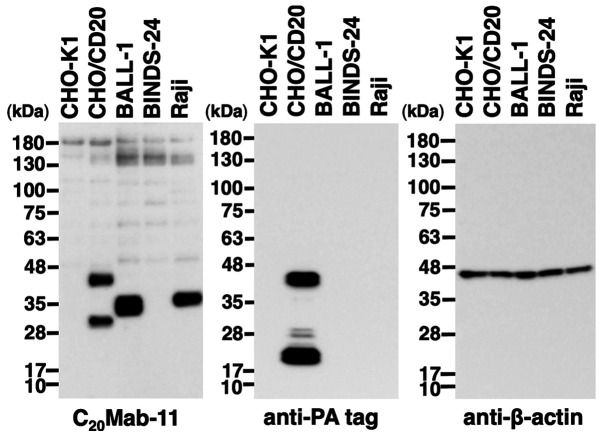
Detection of CD20 by C_20_Mab-11 by western blotting. Cell lysates of CHO-K1, CHO/CD20, BALL-1, BINDS-24 and Raji cells were electrophoresed and transferred onto PVDF membranes. These membranes were treated with C_20_Mab-11 (left panel), NZ-1 (anti-PA tag; middle panel) or anti-β-actin (right panel), followed by incubation with secondary antibodies.

Next, we performed western blot using C_20_Mab-11. C_20_Mab-11 detected CD20 with a 45-kDa band in CHO/CD20 and a 37-kDa band in BALL-1 and Raji cells; it did not detect CD20 in CHO-K1 cells and CD20-knockout BALL-1 (BIND-24) cells (Fig. 3), indicating that C_20_Mab-11 is specific for CD20. An anti-PA tag mAb (NZ-1) detected CD20 at a 45-kDa band without detecting any bands in CHO-K1, BALL-1, BINDS-24, and Raji cells. The difference of molecular size between CHO/CD20 and BALL-1 cells might be due to N-terminal PA-tag of CD20. Although additional bands of 30 and 20 kDa were detected by C_20_Mab-11 and an anti-PA tag mAb (NZ-1), respectively, in CHO/CD20 cells, we postulated that they might be degraded fragments of the CD20 protein.

#### Immunohistochemical analyses

**Figure 4.**
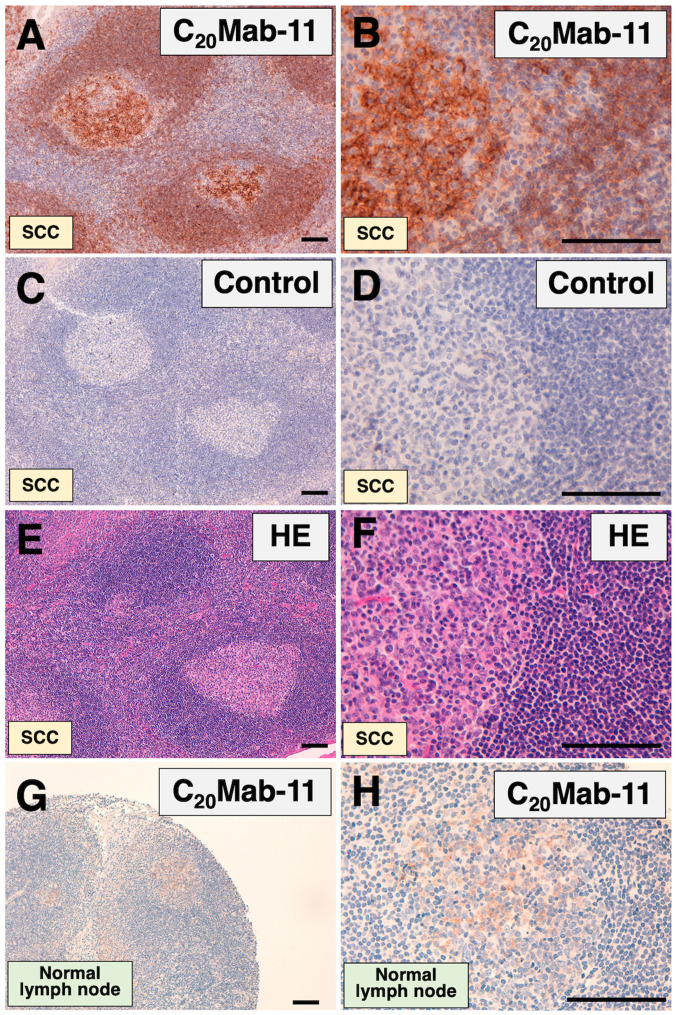
Immunohistochemical analyses using C_20_Mab-11 for OSCC or normal lymph node. (A and B) Consecutive tissue sections of OSCC were incubated with C_20_Mab-11, followed by an Envision+ kit. Counterstaining was performed using hematoxylin. (C and D) Consecutive tissue sections of OSCC were incubated with blocking buffer, followed by an Envision+ kit. Counterstaining was performed using hematoxylin. (E and F) HE staining was also performed using consecutive OSCC tissues. (G and H) Consecutive tissue sections of normal lymph node tissue were incubated with C_20_Mab-11, followed by an Envision+ kit. Counterstaining was performed using hematoxylin. Scale bar, 100 µm. OSCC, oropharyngeal squamous cell carcinoma; HE, hematoxylin and eosin.

We further investigated the immunohistochemical utility of C_20_Mab-11 in a lymph node of oropharyngeal squamous cell carcinoma patient. C_20_Mab-11 strongly stained the lymph follicle and weakly stained the cortex (Fig. 4A and B). No staining was observed without the primary antibody (Fig. 4C and D). Hematoxylin and eosin (HE) staining was also performed using consecutive oropharyngeal squamous cell carcinoma tissues (Fig. 4E and F).

**Figure 5.**
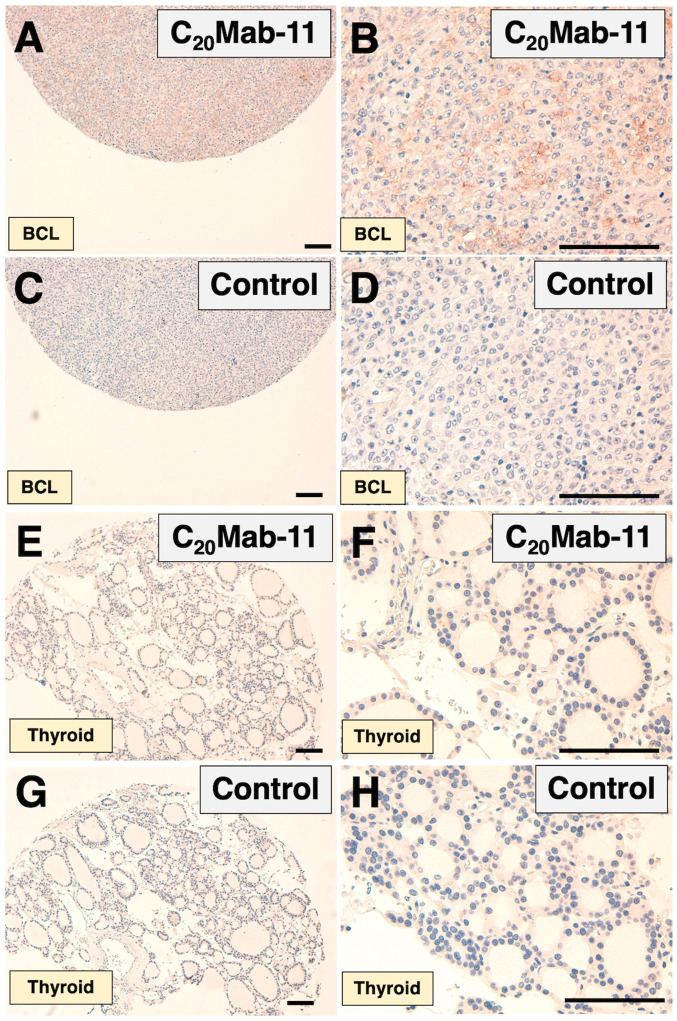
Immunohistochemical analyses using C_20_Mab-11 for B-cell lymphomas and B-cells. (A and B) Consecutive tissue sections of BCL were incubated with C_20_Mab-11, followed by an Envision+ kit. Counterstaining was performed using hematoxylin. (C and D) Consecutive tissue sections of BCL were incubated with blocking buffer, followed by an Envision+ kit. Counterstaining was performed using hematoxylin. (E and F) Consecutive tissue sections of thyroid were incubated with C_20_Mab-11, followed by an Envision+ kit. Counterstaining was performed using hematoxylin. (G and H) Consecutive tissue sections of thyroid were incubated with blocking buffer, followed by an Envision+ kit. Counterstaining was performed using hematoxylin. Scale bar, 100 µm.

C_20_Mab-11 weakly stained B cells of normal lymph node (Fig. 4G and H) and B cell-lymphomas (Fig. 5A and B). No staining was observed in B cell-lymphomas without the primary antibody (Fig. 5C and D). C_20_Mab-11 (Fig. 5E and F) and control (Fig. 5G and H) did not stain normal thyroid tissues. These results indicate that C_20_Mab-11 is useful for detecting B cells in immunohistochemical analyses using FFPE tissues.

## Discussion

In this study, we successfully established a sensitive and specific anti-CD20 mAb, C_20_Mab-11, using the CBIS method, previously developed by our laboratory (Fig. 1) (7). Because this CBIS method does not need purified proteins for immunization and screening, we can develop mAbs against multi-pass transmembrane proteins more effectively. Previously, we developed anti-CD133 mAbs using CBIS method without purifying CD133 proteins (7). The anti-CD133 mAbs were determined to be useful for every application, including for flow cytometry, western blot, and immunohistochemical analyses. While there are several anti-CD133 mAbs are commercially available, the applications of those mAbs are usually limited (19); therefore, we have to use several mAbs or polyclonal antibodies for different experimental procedures.

We can also obtain many anti-CD20 mAbs commercially as shown in Table SI. As listed, anti-CD20 mAbs are usually applicable for only one or two experimental techniques. Although a few mAbs are useful for at least three applications, including flow cytometry, they do not react with the extracellular domains of CD20 because only the intracellular domains were used as the immunogen. Rituximab [a mouse-human chimeric mAb; the original mouse clone is 2B8 (20)] is known as the first anti-CD20 mAb approved by the US FDA and is used for the treatment of B-cell NHL or B-cell chronic lymphocytic leukemia (21). Rituximab shows high sensitivity in flow cytometry as well as exerting high antibody-dependent cell-mediated cytotoxicity and complement dependent cytotoxicity (ADCC/CDC) and anti-tumor activities. However, rituximab could not detect CD20 protein in western blot analysis in our study (data not shown). In contrast, C_20_Mab-11 could detect endogenous CD20 protein, which is expressed in BALL-1 and Raji cells, very sensitively (Fig. 3). Furthermore, C_20_Mab-11 could stain normal B-cells or B-cell lymphoma cells in immunohistochemical analyses using FFPE tissues (Figs. 4 and 5). Because mAbs possess different epitopes, it might be preferable to use the same mAb of the accordant epitope for both diagnosis and therapy. In this study, we used one oropharyngeal squamous cell carcinoma tissue, one B-cell lymphoma tissue, and two normal human tissues for C_20_Mab-11 immunostaining. In our future study, it would be useful to further validate our results in more tissues and with different degrees of disease progression.

C_20_Mab-11 could be also advantageous for targeting CD20-expressing B-cell lymphomas. Our future studies will focus on changing the subclass of C_20_Mab-11 (mouse IgM) into mouse IgG_2a_ or human IgG_1_ and determine whether these mAbs also demonstrate ADCC/CDC and anti-tumor activities (22).

Anti-CD20 mAbs in combination with rituximab, ^131^I-Tositumomab (radio-immunotherapy) (23), ibritumomab tiuxetan (radio-immunotherapy) (24), ofatumumab (a fully human mAb) (25), or obinutuzumab (a humanized glycoengineered mAb) (26), have brought significant survival benefits to B-cell lymphoma patients. In follicular lymphoma, the four-year overall survival rate was significantly improved with the combination of cyclophosphamide, doxorubicin, vincristine, prednisone (CHOP) therapy and an anti-CD20 mAb (91%) compared with CHOP alone (69%) (27). In elderly patients (60–80 years old) with diffuse large B-cell lymphoma, overall survival was improved by 13% following treatment with rituximab plus CHOP compared with CHOP alone (27). Although anti-CD20 mAbs have been shown to be very effective for the treatment of B-cell lymphoma, many patients do not have a clinical response to anti-CD20 mAbs (28). Furthermore, 60% of NHL relapse patients, who initially responded to rituximab, have no subsequent clinical response to rituximab due to the loss of CD20 expression (28). To overcome resistance to anti-CD20 mAbs, the development of more effective first-line lymphoma treatments is needed. Future studies will investigate whether C_20_Mab-11 may be used as an antibody-containing drug to treat CD20-expressing lymphomas.

## Supplementary Material

Supporting Data

## Data Availability

The datasets used and/or analyzed during the study are available from the corresponding author on reasonable request.

## Abbreviations

mAb: monoclonal antibody
CBIS: Cell-Based Immunization and Screening
BSA: bovine serum albumin
DAB: 3,3′-diaminobenzidine tetrahydrochloride
