# Supplementary material for: Establishment of C20Mab-11, a novel anti-CD20 monoclonal antibody, for the detection of B cells
Source: Oncol Lett. 2020 Jun 17;20(2):1961–7. doi: 10.3892/ol.2020.11753 (PMC7377059; doi:10.3892/ol.2020.11753)
Supplement: Supporting Data [file Supplementary_Data.pdf]

Table SI. Commercially available anti-CD20 mAbs.

Clone	Company	Species	Isotype	Immunogen	Application		
					FCM	IHC	WB
4A7G3	ProteinTech Group, Inc.	Mouse	IgG_{2b}	Protein	+ (i.c.)	+	+
EP459Y	Abcam	Rabbit	IgG	Peptide (i.c.)	+ (i.c.)	+	+
SP32	Abcam	Rabbit	IgG	Peptide (i.c.)	+ (i.c.)	+	+
L26	Abcam	Mouse	IgG_{2a}	B cells	+ (i.c.)	+	+
MS4A1/3409	Abcam	Mouse	IgG_{2b}	Peptide (i.c.)	+ (i.c.)	+	+
MEM-269	GeneTex, Inc.	Mouse	IgM	RAMOS cells	+	+	-
SPM618	GeneTex, Inc.	Mouse	IgG_{2a}	Protein	+	+	-
IGEL/1497R	GeneTex, Inc.	Rabbit	IgG	Protein	+	+	-
E7B7T	Cell Signaling Technology, Inc.	Rabbit	IgG	Protein	-	+	+
UMAB37	OriGene Technologies, Inc.	Mouse	IgG_1	Protein	-	+	+
UMAB38	OriGene Technologies, Inc.	Mouse	IgG_1	Protein	-	+	+
UMAB39	OriGene Technologies, Inc.	Mouse	IgG_1	Protein	-	+	+
UMAB58	OriGene Technologies, Inc.	Mouse	IgG_1	Protein	-	+	+
OTI1H4	OriGene Technologies, Inc.	Mouse	IgG_1	Protein	-	+	+
OTI2C11	OriGene Technologies, Inc.	Mouse	IgG_{2b}	Protein	-	+	+
OTI3C4	OriGene Technologies, Inc.	Mouse	IgG_1	Protein	-	+	+
OTI4A4	OriGene Technologies, Inc.	Mouse	IgG_1	Protein	-	+	+
OTI4B4	OriGene Technologies, Inc.	Mouse	IgG_1	Protein	-	+	+
OTI10A5	OriGene Technologies, Inc.	Mouse	IgG_1	Protein	-	+	+
OTI11F7	OriGene Technologies, Inc.	Mouse	IgG_1	Protein	-	+	+
MEM-97	Abcam	Mouse	IgG_1	Raji cells	+	-	-
B-Ly1	Abcam	Mouse	IgG_1	not available	+	-	-
2H7	Abcam	Mouse	IgG_{2b}	B cells	+	-	-
B9E9	Abcam	Mouse	IgG_{2a}	Daudi cells	+	-	-
B-H20	Abcam	Mouse	IgG_{2a}	CLL cells	+	-	-
GT0008	GeneTex, Inc.	Mouse	IgG_{2a}		+	-	-
LT20	GeneTex, Inc.	Mouse	IgG_{2a}	Lymphocytes	+	-	-
ICO-180	Thermo Fisher Scientific, Inc.	Mouse	IgG_1	B cells	+	-	-
743AB35	OriGene Technologies, Inc.	Mouse	IgG_{2a}	cDNA	+	-	-
743AB71	OriGene Technologies, Inc.	Mouse	IgG_{2a}	cDNA	+	-	-
743X45	OriGene Technologies, Inc.	Mouse	IgG_{2a}	cDNA	+	-	-
743X56	OriGene Technologies, Inc.	Mouse	IgG_{2b}	cDNA	+	-	-
743X69	OriGene Technologies, Inc.	Mouse	IgG_{2a}	cDNA	+	-	-
743X78	OriGene Technologies, Inc.	Mouse	IgG_{2a}	cDNA	+	-	-
743X65	OriGene Technologies, Inc.	Mouse	IgG_{2a}	cDNA	+	-	-
IGEL/773	Abcam	Mouse	IgG_{2a}	Protein	-	+	-
3E9D3C1G3	GeneTex, Inc.	Mouse	IgG	Peptide	-	+	-
OTI1C12	OriGene Technologies, Inc.	Mouse	IgG_1	Protein	-	-	+

i.c., intracellular; FCM, flow cytometry; IHC, immunohistochemistry; WB, western blot.
